# Polysaccharide Fraction Extracted from Endophytic Fungus *Trichoderma atroviride* D16 Has an Influence on the Proteomics Profile of the *Salvia miltiorrhiza* Hairy Roots

**DOI:** 10.3390/biom9090415

**Published:** 2019-08-26

**Authors:** Wei Peng, Qian-liang Ming, Xin Zhai, Qing Zhang, Khalid Rahman, Si-jia Wu, Lu-ping Qin, Ting Han

**Affiliations:** 1 Department of Pharmacognosy, School of Pharmacy, Second Military Medical University, 325 Guohe Road, Shanghai 200433, China; 2 School of Pharmacy, Chengdu University of Traditional Chinese Medicine, No.1166 Liutai Avenue, Chengdu 611137, China; 3 Department of Pharmacognosy, School of Pharmacy, Army Medical University, 30 Gaotanyan Street, Chongqing 400038, China; 4 Faculty of Science, School of Pharmacy and Biomolecular Sciences, Liverpool John Moores University, Byrom Street, Liverpool L3 3AF, UK; 5 School of Pharmacy, Zhejiang Chinese Medical University, Hangzhou 310053, China

**Keywords:** polysaccharide fraction, *Trichoderma atroviride*, *Salvia miltiorrhiza*, proteomics, tanshinones

## Abstract

*Trichoderma atroviride* develops a symbiont relationship with *Salvia miltiorrhiza* and this association involves a number of signaling pathways and proteomic responses between both partners. In our previous study, we have reported that polysaccharide fraction (PSF) of *T. atroviride* could promote tanshinones accumulation in *S. miltiorrhiza* hairy roots. Consequently, the present data elucidates the broad proteomics changes under treatment of PSF. Furthermore, we reported several previously undescribed and unexpected responses, containing gene expression patterns consistent with biochemical stresses and metabolic patterns inside the host. In summary, the PSF-induced tanshinones accumulation in *S. miltiorrhiza* hairy roots may be closely related to Ca^2+^ triggering, peroxide reaction, protein phosphorylation, and jasmonic acid (JA) signal transduction, leading to an increase in leucine-rich repeat (LRR) protein synthesis. This results in the changes in basic metabolic flux of sugars, amino acids, and protein synthesis, along with signal defense reactions. The results reported here increase our understanding of the interaction between *T. atroviride* and *S. miltiorrhiza* and specifically confirm the proteomic responses underlying the activities of PSF.

## 1. Introduction

*Salvia miltiorrhiza* is a Chinese traditional medical herb widely used for preventing and treating disorders of liver, vascular, menstrual, and blood circulation systems. Diterpenoid pigments are the main bioactive constituents of *S. miltiorrhiza* roots, and exert anti-inflammatory properties via significant inhibition of production of NO, IL-1β, and TNF-α [1,2]. In particular, tanshinones, the predominant active constituents in the roots of *S. miltiorrhiza*, possess various promising bioactivities, such as anti-inflammation, anticoagulation, and liver protection, etc. [2,3]. However, the current production of tanshinones is not meeting the current medicinal market needs. Hairy root can yield a great number of secondary metabolites due to its fast growth and biochemical stability. As a typical material in plant science research, *S. miltiorrhiza* hairy roots were investigated for increased production of diterpenoid constituents for pharmaceutical usages.

Endophytic fungi, as non-harm microbes, can form long-term beneficial relationships with the host plant and could also produce some active natural secondary metabolites mainly including small molecules, which can be explained by the hypothesis of balance antagonism. Endophytic fungi is distinctive from normal chemical elicitors with the same effects on secondary metabolites accumulation as biotic inducer as reported [4,5,6]. Endophytic fungi can break through the first defense line of plants’ microbe-associated molecule patterns (MAMPs) [7], and the cell wall is the primary structure of plants that comes in contact with its microbes. Chitin has been identified and studied as a well-known elicitor in pathogens or beneficial fungi; and great importance is also attached to β-glucan in microbe–plant relationship research. As previously reported, polysaccharides are another type of elicitors in the cell walls of fungus and bacterium to induce the resistance of plants, as well as promoting the accumulation of secondary metabolites [7,8]. Consequently, chitins, β-glutan, and polysaccharides are the components of the fungal cell wall [9]; although Chen et al. [10] have implicated that mannose can be listed as a new elicitor which is different from other known elicitors, thus broadening our knowledge and perception of other components of the fungi cell wall as elicitors [11]. In our previous study, we found that the *Trichoderma atroviride*, an endopyhtic fungus isolated from *S. miltiorrhiza*, was able to produce tanshinone I and tanshinone IIA. In addition, we also reported that the extracts of mycelium (EM) and the polysaccharide fraction (PSF) of *T. atroviride* have more efficient influences than live fungus, especially the PSF. Therefore, PSF may be the key active constituent as elicitor of *T. atroviride* in eliciting tanshinones accumulation [6].

Though the balance antagonism hypothesis can explain the interaction mechanism between endophytic fungi and host plant, the molecular signaling transduction process of plants in response to endophytic fungi is still unclear [12]. Due to post-translational events and modifications, proteomics is more specific compared to mRNA when investigating signaling and metabolic processes in plants [13,14]. Currently, the isobaric tags for relative and absolute quantitation (iTRAQ) have developed into significant technology which can analyze, quantify, and compare protein levels linked with liquid chromatography-quadrupole mass spectrometry (LC-MS/MS), providing greater efficiency and accuracy when compared with gel-based techniques. Some reports of novel differently-expressed types of proteins in the interaction between host plant and endophytic fungi do exist [15,16]. For example, the endophytic fungus *Gilmaniella* sp. AL12 can induce *Atractylodes lancea* to yield volatile oils through G protein-mediated signal transduction and the mannose-binding lectin pathway according to proteome analysis [17]. The aim of the present study was to investigate the influences of PSF from *T. atroviride* D16 (D16 PSF) on the proteomics profile of the *S. miltiorrhiza* hairy roots and its possible mechanisms based on iTRAQ strategy.

## 2. Materials and Methods

### 2.1. The Culture and Treatment of Hairy Root

Simple hairy roots were separated into different 250 mL conical flasks equipped with 100 mL half-strength B5 liquid medium at 25 °C, 135 rpm in the dark. Every Erlenmeyer flask was loaded with 0.1 g hairy root and was cultured for 3 weeks and the liquid medium was changed weekly. The blank and the treatment group were cultured in half B5 liquid medium, except that the treatment group also contained 60 mg/L PSF. The hairy root samples were taken out for further experiments on the third and sixth days after treatment commenced.

The samples were handled with liquid nitrogen to remove the moisture and then deposited in −80 °C refrigerator. The blank group samples taken on day 3 were numbered A1 and A2, while treatment group samples were numbered B1 and B2. Similarly, the blank group samples taken on day 6 were numbered C1 and C2, while treatment group samples were numbered D1 and D2. However, hairy root samples were disposed by the same method on the twelfth day after treatment for RNA extraction.

### 2.2. The Preparation of PSF

The hyphae of *T. atroviride* D16 was transferred into the newly-made Potato Dextrose Agar (PDA) medium for 4 days and then moved into 250 mL conical flasks containing 100 mL half B5 liquid medium and cultured for 3 days. Finally, the cultured D16 was added into 5 L conical flask for amplified culturing between 7 to 10 days. Subsequently, the mycelia were collected with vacuum suction filtration, washed three times with distilled water and homogenized by placing in a blender for 10 min, followed by addition of three volumes of distilled water equivalent to the wet weight of mycelia. The water solution of mycelia was then heated to a temperature of 121 °C under high pressure for 60 min and was filtered as fungal elicitor. The filtrate was decompressed and concentrated to an appropriate volume at 75 °C and mixed with 4 times volume of 95% ethanol for 2 days at room temperature. After lyophilization, the precipitate was further subjected to deproteinization with Sevag reagent (chloroform:n-butanol, 4:1, *v*/*v*), and small molecule impurities were removed as well as proteins less than 2000 kDa. Thereafter, the solution was freeze-dried under vacuum into powder as PSF.

### 2.3. Monosaccharides Composition of PSF

A total of 5.00 mg D16 PSF was hydrolyzed with trifluoroacetic acid (TFA, 2 M) for 2 h at 120 °C hermetically, the TFA was then evaporated under vacuum, and the residue was subsequently washed three times with methanol. The residue was then mixed with 30 mg NaBH_4_ and 1mL H_2_O and left overnight for reduction followed by the addition of methanol and acetic acid at a ratio of 5:1, the solvent was evaporated under reduced pressure, followed by the addition and evaporation of methanol three times. The sample was then left to dry at 105 °C for 10 min followed by acetylation of the sample by the addition of 3 mL acetic anhydride (AC_2_O), for 60 min at 105 °C, after which the reaction was terminated by the addition of 1 mL water followed by vortex mixing. The acetylation products were extracted with 2 mL chloroform, which was then dried by the addition of anhydrous sodium sulfate prior to GC-MS analysis. The standard products (including rhamnose, arabinose, xylose, fructose, mannose, glucose, and galactose, which were purchased from the National Institute for Food and Drug Control, Beijing, China) were subjected to the same procedure as the sample without hydrolyzation.

GC-MS analysis conditions: The size of TR-5MS (Thermo) chromatographic column was 60 m × 0.25 mm × 0.25 mm; initial conditions: The programmed temperature was 140 to 198 °C at 2 °C/min and kept for 4 min. The temperature was then increased to 214 °C at 4 °C/min and 217 °C at 1 °C/min for 4 min. Finally, the temperature was raised to 250 °C at 3 °C/min for 5 min; injection port temperature was 250 °C; the carrier gas was He; the flow rate was 1 mL/min; ion source 250 °C; *m*/*z* = 40-500; EI = 70 ev.

### 2.4. HPLC Analyses

The hairy roots were dried at 50 °C in an oven until a constant weight was obtained and then grounded into powder form. The powder was then extracted with chromatographic pure methanol for 60 min under sonication. Next, the methanol extract was applied to the high-performance liquid chromatography (HPLC) system-Agilent-1100-for analysis. The Agilent-1100 was carried out with a H_2_O (containing 0.5% HCOOH) (A)/acetonitrile (B) gradient by a ZORBAX SB-C_18_ chromatographic column (250 × 4.6 mm, 5 μm) at 30 °C [6]. The reference standards of dihydrotanshinone I (DT-I), tanshinone I (T-I), cryptotanshinone (CT), and tanshinone IIA (T-IIA) were purchased from the Chengdu Mansite Pharmaceutical Co. Ltd. (Chengdu, China). In addition, dihydrotanshinone I (0.0025, 0.0050, 0.0100, 0.0200, and 0.0400 mg/mL), tanshinone I (0.002, 0.004, 0.006, 0.008, and 0.010 mg/mL), cryptotanshinone (0.001, 0.005, 0.010, 0.025, 0.050, and 0.100 mg/mL), and tanshinone IIA (0.0005, 0.001, 0.002, 0.004, 0.006, and 0.008 mg/mL) were used to prepare the standards curves. The methanol extract of hairy roots was analyzed and the peaks identified and contrasted in comparison with the available standards.

### 2.5. RNA Isolation and Real-Time Quantitative PCR Analysis

TRIzol reagent (Invitrogen Co., Carlsbad, CA, USA) was used to extract the total RNA of hairy root and 1 µL RNA was diluted to 100 µL with RNase ddH_2_O. According to the RNA concentration ratio of 260 and 280 nm UV absorbance value, OD260/280 should be between 1.9 to 2.1 and the total RNA concentration was calculated according to the formula total RNA (µg/mL) = OD260 × 100 (dilution multiple) × 40 µg/mL. The reverse transcription reaction was conducted at 37 °C for 15 min, 85 °C for 5 s termination reaction at ABI 9700 PCR. Finally, 90 µL RNase-free ddH_2_O was added to 100 µL for storing at −20 °C for Real-time PCR. Primers with the following sequences, LRRK F (5′-TGTGGTAGCTTTGTGGGGTT-3′) and LRRK R (5′-CAGACCGGAGATTGAGTCCG-3′), LRRK-PEPR2 F (5′-TGTGGTAGCTTTGTGGGGTTT-3′) and LRRK-PEPR2 R (5′-GCCAGACCGGAGATTGAGTC-3′), Nucleoporin F (5′-GTCAAAACCTGCAACCACCT-3′) and Nucleoporin R (5′-AGAATGCTGGAGAAATGCCG-3′), probable cation-transporting ATPase F (5′-TGTCCCCATGAATTAGAACTGGT-3′) and probable cation-transporting ATPase R (5′-TGGCGACTTTTGCAGTCAAC-3′), primers identifying the Smactin gene, Smactin F (5′-ATGATAACTCGACGGATCGC-3′) and Smactin R (5′-CTTGGATGTGGTAGCCGTTT-3′), were used as a reference gene to normalize cDNA loading. The real-time PCR amplification was performed in a 384-well plate Roche LC480 thermocycler (Roche Diagnostic Basel, Switzerland) with Super Real PreMix kit (TIANGEN, Beijing, China). Each reaction contained a mixture of 1 μL of diluted cDNA, 0.2 μL of forward primer (10 μM), 0.2 μL of reverse primer (10 μM), 5 μL of SYBR Green PCR Master Mix (TIANGEN, Beijing, China), and 3.6 μL of RNase-free H_2_O. The reaction mixture was incubated for 15 min at 95 °C, and for 40 cycles of 10 s at 95 °C and 30 s at 60 °C. The relative gene expression was quantified using the comparative CT method.

### 2.6. Protein Extraction of Salvia Miltorrhiza Hariy Root

A total of 0.5000 g of *S. miltiorrhiza* hairy root sample was grounded into powder in liquid nitrogen and transferred into 10% trichloroacetic acid and precooling acetone solution containing 65 mM Dithiothreitol (DTT). Followed precipitation at −20 °C for 6 h, the sample was centrifuged at 10,000 rpm, at 4 °C for 45 min, and the sediment was re-suspended by the addition of PH standard reagent (STD buffer) according to the volume ratio of 10:1. The sample was then vortexed and blended and placed in a boiling water bath for 5 min and then subjected to ultrasound 10 times (ultrasound for 10 s, interval for 15 s). Finally, the sample was heated in a water bath for 5 min and subjected to centrifuging for 15 min at 10,000 rpm. The supernatant containing the total protein extract of *S. miltiorrhiza* hairy root was then stored at −80 °C until assayed for protein content by the bicinchonininc acid (BCA) and Bradford method.

### 2.7. The Mass Spectrum Analysis of Protein

The protein samples were enzymolyzed and the peptides solution was qualified by measuring at OD280. The sample were then analyzed by LC-MS preliminarily and performed on AKTA Purifier 100 (GE Healthcare, Chicago, IL, USA) for SCX classification via strong cation chromatographic column. Finally, the samples were separated by the Easy nLC liquid phase system of the nanoliter velocity. Q-Exactive mass spectrometer (Thermo Finnigan, Silicon Valley, CA, USA) was used to analyze the mass spectra of the sample solution after capillary HPLC separation. The analysis time was 120 min, testing method was positive ion mode, and the mother ion scanning ranged from 300 to 1800 *m*/*z*. The level of mass spectrum resolution was 70,000 (*m*/*z* 200), AGC target was 3e6, first level of Maximum IT was 10 ms, number of scan ranges was 1, and dynamic exclusion was 40 s. The ion-charge ratio of peptide and the peptide fragments was collected according to the following methods: Full scan acquiring 10 secondary debris each map; secondary excitation types: heated capillary dissociation (HCD); separate window: 2 *m*/*z*; secondary mass spectrum resolution: 17,500 (200) *m*/*z*; microscans: 1; secondary maximum IT: 60 ms; standard collision energy: 30 eV; under fill the wire: 0.1%.

### 2.8. Data Analysis

The raw data of mass spectrometry was analyzed with software Mascot 2.2 and the identified proteins were blasted with UniProt database for the Gene Ontology (GO) classification annotation. The peak strength value of the reported ion was analyzed quantitatively with Proteome Discoverer1.4 software and the differentially expressed genes of Fc (fold change, Fc (B/A) = B/A, Fc (D/C) = D/C) were screened according to the standard of Fc ≥ 1.50 or Fc ≤ 0.66. The differentially expressed proteins identified in the Arabidopsis database were compared to the database (UniProt, SWISSPROT and TREMBL) by using the software BLASTP (2.2.23+) and the comparison results were extracted. These comments were corresponded to the GO number by the gene association file (gene association. Goa uniprot) and the number of the corresponding small items were counted in the three large catalogs of the biological process, cellular component, and molecular function. According to the bi-directional best hits (BBH) analysis, the corresponding number was mapped to KEGG pathway maps and the total protein of Arabidopsis was calculated by using the hyper-geometric distribution of *p*-value. The false discovery rate (FDR) was then used to correct it and z-score was calculated at the same time. The pathway of significant enrichment was the pathway of FDR is less than 0.05 and z-score > 0. INTACT and String was used to construct differentially expressed protein interaction networks: (a) Searching INTACT to build network (http://www.ebi.ac.uk/intact/); (b) using the software String to build the network (http://string.embl.de).

All experiments, including both control and different treatments of hairy root cultures, HPLC analysis, and semi-quantitative real-time PCR, were performed in triplicate. The results are presented as their mean values and standard deviations (SD). The error bars in the figures represent the standard deviation in biological triplicates. The statistical significance of the differences in root growth and the accumulation of phenolic acids and tanshinones were analyzed by one-way analysis of variance (ANOVA) with SPSS software (version 18.0, Chicago, IL, USA), the heatmap was drawn using Excel software (version: 2010, Microsoft Co., Redmond, WA, USA), and the term significant was used to denote the differences for which *p*-value is < 0.05. The statistical significance of differences in gene transcripts was analyzed by one-sample *t*-test.

## 3. Results

### 3.1. Monosacharide Composition and Its Effects on Accumulation of Tanshinones of the D16 PSF

**Figure 1:**
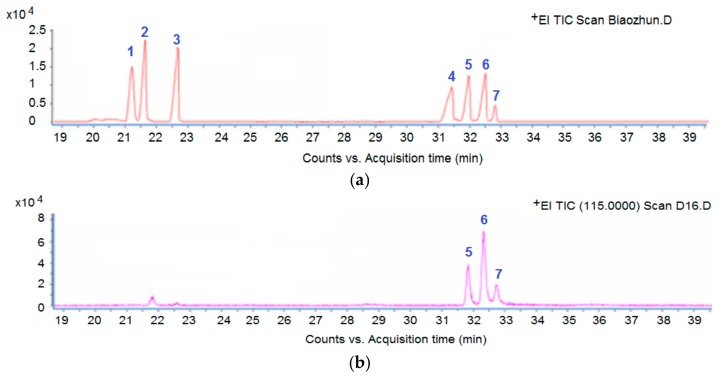
GC-MS analysis of D16 PSF monosaccharides components. (**a**) GC-MS chromatogram of monosaccharide standards; (**b**) GC-MS chromatogram of the D16 PSF sample. The numbers 1 to 7 represented the rhamnose, arabinose, xylose, fructose, mannose, glucose, and galactose, respectively. D16 PSF means polysaccharide fraction from *T. atroviride* D16.

D16 PSF was extracted from *T. atroviride* D16 and it had greatly induced the accumulation of tanshinones in *S. miltiorrhiza* hairy roots, as reported in the previous study [6]. To further determine the effects of hydrolytic constitutes on *S. miltiorrhiza* hairy root, the D16 PSF was hydrolyzed to determine its monosaccharides compositions, and the results revealed that D16 PSF was composited by mannose, glucose, and galactose at a ratio of 5:10:2 (Figure 1). The standard samples of mannose, glucose, and galactose were made into the solution with the concentration of 60 mg/L, respectively, and then put into the *S. miltiorrhiza* hairy root culture medium in triplet for 14 days. The results showed the content of tanshinones in *S. miltiorrhiza* hairy root after 14 days’ culture varied in the presence of PSF and the monosaccharides (Table 1). Compared with the control group, PSF (60 mg/L) stimulated the tanshinones accumulation at the greatest extent. Different components of PSF had various effects on promoting synthesis of tanshinones; however, the singular result of each D16 PSF constitute could not catch up with the influence of PSF on the accumulation of tanshinones. Under the treatment of PSF (60 mg/L), the content of dihydrotanshinone I was similar to the results at the same concentration of mannose and galactose respectively, while the equally-increased effects on tanshinone I were among the different treatments of PSF (60 mg/L), glucose, and mannose. Under the treatment of PSF (60 mg/L), the content of cryptotanshinones was 3.08-fold higher than the control group, while less increased effects occurred followed by the treatment of glucose. Galactose made the cryptotanshinones content decreased, while mannose had no effect on the accumulation of cryptotanshinones.

### 3.2. Differential Proteomics of S. miltiorrhiza in Response to D16 PSF

**Figure 2:**
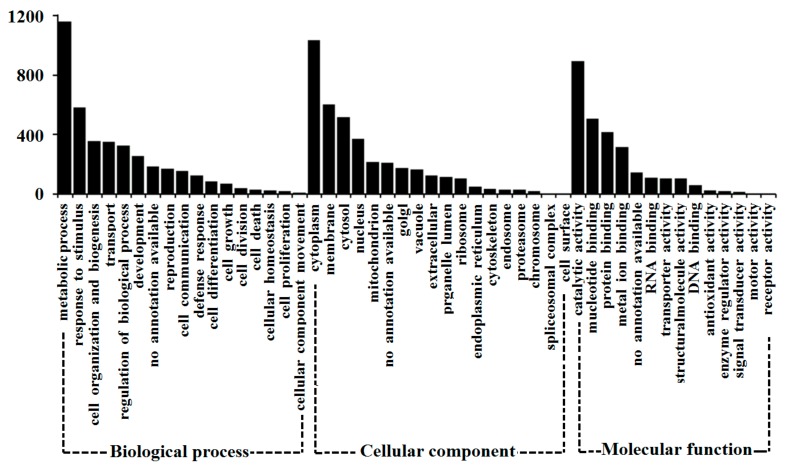
Gene ontology classification of the proteins identified base on *Arabidopsis thaliana* database.

The differences of protein content in *S. miltiorrhiza* hairy roots treated with D16 PSF were studied by proteomics technique of iTRAQ. Based on the database of *Arabidopsis thaliana*, 3685 feature peptides (unique peptide) belonged to 1476 proteins, which were identified and analyzed for GO classification annotation. As shown in Figure 2, most of proteins can be annotated into the three ontologies of biological process, cellular component, and molecular function. In the biological process annotation, the number of proteins in the metabolic process (1162) and response to stimulus (584) was the largest, and 187 proteins were not annotated. In cellular components annotation, positioning to the cytoplasm (1036), the cell membrane (602), and the cytosol (517) account for the greatest number of proteins, while 211 proteins had not been annotated. In the molecular function annotation, a large number of proteins were divided into catalytic activity (894), nucleotide binding (507), protein binding (417), as well as metal ion binding (315) under the entry and 146 proteins had not been annotated.

**Figure 3:**
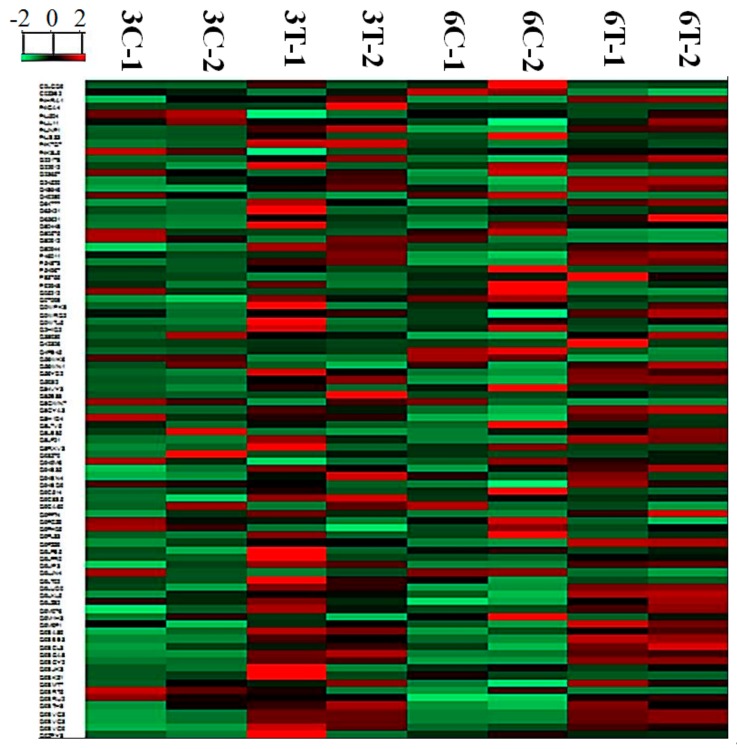
Heatmap of statistically-changed proteins identified based on *Arabidopsis thaliana* database. High- and low-expression are shown in green and red; C and T indicate control and PSF-treated, respectively.

**Figure 4:**
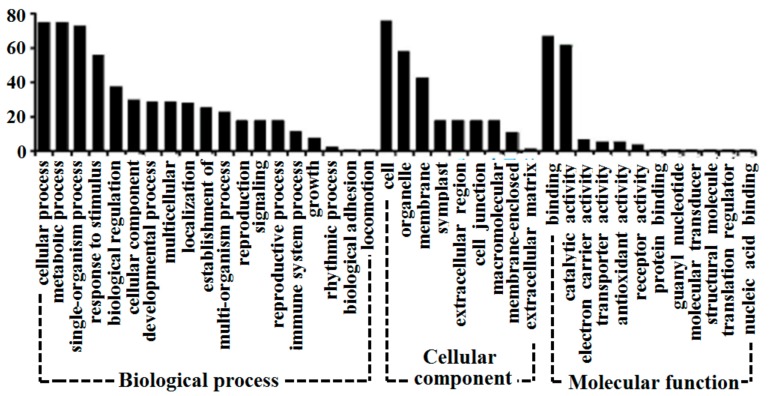
Gene ontology classification of the differential proteins identified based on *Arabidopsis thaliana* database.

**Figure 5:**
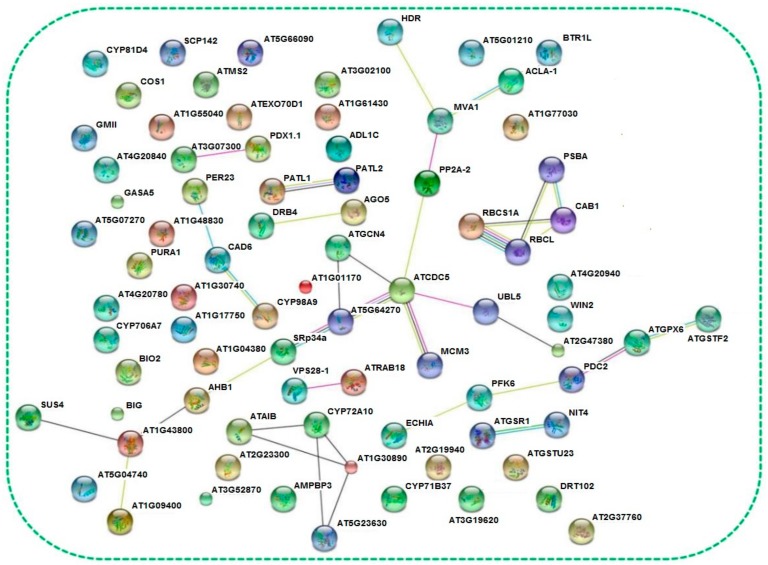
String network of the differential proteins identified based on *Arabidopsis thaliana* database.

Among the 1476 proteins, 89 differentially expressed proteins were picked out for quantitative analysis (Figure 3). The classification of 89 different proteins was identified and analyzed in the *Arabidopsis thaliana* database. In addition, the number of proteins was corresponded to each category, as shown in Figure 4. The 89 proteins corresponded to 39 KEGG pathways, including amino acid metabolism, sugar metabolism, energy metabolism, and secondary metabolites synthesis. The first 10 KEGG pathways were annotated as shown in Table 2. The 89 different proteins were analyzed by adopting the INTACT and String interaction networks and the results are shown in Figure 5.

### 3.3. Quantitative Analysis of the 89 Differential Proteins

**Figure 6:**
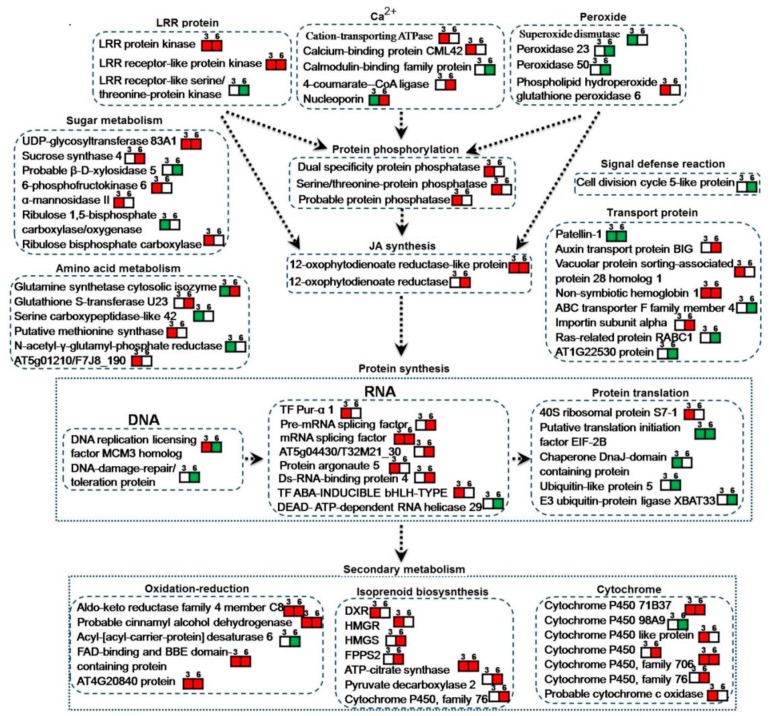
The action network map of 89 differential proteins according to the *Arabidopsis thaliana* database. The number in the box indicates the number of PSF-treated days, red indicated significant increase and green indicated significant reduction.

For the quantitative analysis, 89 differential proteins were mainly involved in signal transduction, redox, amino acid synthesis and metabolism, protein synthesis and metabolism, the biosynthesis of terpenoids, carbohydrate synthesis, metabolism and transport, and other physiological and biochemical process (Table 3, Figure 6).

**Figure 7:**
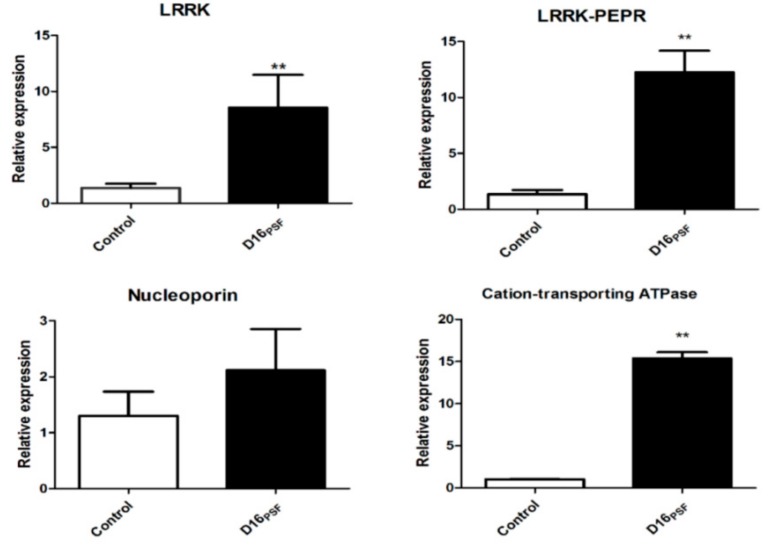
Relative expression of leucine-rich repeat protein kinase (LRRK), leucine-rich repeat protein kinase PEPR (LRR-PEPR), cation-transporting ATPase, and nucleoporin after D16 PSF treatment on the fourteenth day. Values are presented as means ± SD (*n* = 3). * *p* < 0.05; ** *p* < 0.01 versus the control culture.

The 89 differential proteins in signal transduction involves the leucine-rich repeat (LRR) protein, Ca^2+^-related protein, peroxidase-related protein, protein phosphorylation protein, and the synthesis of jasmonic acid (JA). We found that two LRR-related proteins significantly increased leucine-rich repeat protein kinase family protein and leucine-rich repeat receptor-like protein kinase PEPR2 under the action of *T. atroviride* D16PSF on the third day and sixth day in this study, which revealed that the LRR protein plays an important role in promoting *S. miltiorrhiza* hairy root growth and tanshinone synthesis under the action of *T. atroviride* D16 PSF. The relative expression of LRR and LRR-PEPR on the fourteenth day of *S. miltiorrhiza* after culturing in the presence of D16 PSF was also detected. The results revealed that under the sustained action of D16 PSF, a trend showing an increase in LRR and LRR-PEPR is observed (Figure 7). In our study, we also detected that Ca^2+^-related protein expression changed significantly. The cation-transporting ATPase and calcium-binding protein increased significantly at day three and calmodulin-binding protein (AT3g52870/F8J2_40) decreased significantly on the sixth day and the relative expression of cation-transporting ATPase still increased greatly on the fourteenth day (Figure 7). 4-coumarate-CoA ligase-like 10 increased significantly responding to Ca^2+^ on the sixth day and nucleoporin related to the level of intracellular Ca^2+^ decreased on the third day, but increased on the sixth day significantly [18]. These significant changes of Ca^2+^-related proteins showed calcium plays a very important role under the action of *T. atroviride* D16 PSF in *S. miltiorrhiza* hairy root. At the same time, we also found superoxide dismutase, peroxidase, and peroxidase 50 associated with peroxide was lowered significantly. 12-oxophytodienoate reductase is a very important key enzyme in the jasmonic acid (JA) biosynthetic pathway, and two proteins, 12-oxophytodienoate reductase-like protein and 12-oxophytodienoate reductase (At1g04380), related with jasmonic acid were found to be significantly higher in this study [19]. Therefore, the effect of *T. atroviride* D16 PSF on *S. miltiorrhiza* hairy root may also be related to peroxide, protein phosphorylation, and jasmonic acid.

The 89 differential proteins are mainly involved at the levels of DNA, RNA, and protein translation in protein synthesis. At the level of DNA, the protein–DNA replication licensing factor MCM3 appeared significantly decreased after the first significant rise. DNA and protein damage repair/toleration protein expression was significantly lower on the sixth day. At the level of RNA, transcription factor ABA-inducible bHLH TYPE and transcription factor Pur-alpha 1 was raised significantly on the third day; the pre-mRNA splicing factor SF2-like protein and nuclear protein was also significantly increased. At the level of protein translation, 40 S ribosomal protein was raised significantly on the third day and the rest of the proteins related to the protein degradation were significantly lower such as chaperone DNAJ domain containing protein, the ubiquitin-like 5 and E3 ubiquitin protein–protein ligase XBAT33. These changes showed *T. atroviride* D16 PSF played a positive regulatory role in protein synthesis of *S. miltiorrhiza* hairy root at the protein expression level.

In terms of the differential proteins in secondary metabolism, they were mainly involved in the cytochrome P450 enzymes, redox enzyme, and enzymes related to the terpenoid biosynthetic pathway, and most of these proteins increased significantly under the influence of *T. atroviride* D16 PSF. At the same time, significant changes have taken place in some metabolic activities, like synthesis and metabolism of sugar for providing energy, the amino acid synthesis, and metabolism to provide raw materials for protein synthesis and transporters which have transportation function.

With the deep analysis of these differential expression proteins, it can be presumed that D16 PSF regulates the synthesis and metabolism of saccharides and amino acids, the transcription of genes and the translation of protein through the signal transduction pathways involved in leucine-rich repeat proteins, calcium, peroxides, protein phosphorylation, and jasmonic acid (JA) synthesis. This then results in changes of protein expression involved in secondary metabolism and thus induces metabolic profile changes in *S. miltiorrhiza* hairy roots.

## 4. Discussion

It is known that protein is the carrier of gene function and the executor of the life activities. The high-throughput and large-scale proteomics to study time changes of the expression of all the proteins in the cell or tissue can reveal the physiological and biochemical processes in detail [20,21]. At present, proteomics research mainly is based on the separation of the gel and the liquid phase. The methods based on gel separation include two-dimensional electrophoresis proteomics research and fluorescent difference gel electrophoresis (DIGE) proteomics research, mainly through the gel electrophoresis separation of mixed protein samples. After getting various single protein points, we can screen differential proteins according to the dyed color and identify proteins through the MALDI-MS finally. Methods based on the liquid phase include label-free proteomics research, marked iTRAQ/SILAC proteomics research, and SRM/MRM proteomics research, mainly by means of liquid phase to separate mixed protein samples and through the mass spectrometer connected directly with liquid phase for qualitative and quantitative analysis [22]. Proteomics technology has been widely used in plant growth and development, plant adaptation mechanism to biological and abiotic stress, and the interaction mechanism of microbes [23,24]. iTRAQ (isobaric tags for relative and absolute quantitation) technology is a kind of proteomics technology through four or eight kinds of isotope labels specifically to mark amino groups of polypeptides, and then to identify and separate proteins by LC-MS and analyze relative and absolute content of different protein samples according to the isotope intensity [25,26]. This experiment adopted the iTRAQ technology to tag eight *S. miltiorrhiza* hairy root samples of control group and *T. atroviride* D16 PSF on the third and sixth day.

Leucine-rich repeat (LRR) protein kinase family proteins are the largest known type of transmembrane receptor protein kinases in plants, which have the function of regulation in plant growth and development, hormone signal transduction, and biological and non-biological stress response [27,28]. LRR proteins play a critical role in the process of *Piriformospora indica* promoting growth of *Arabidopsis thaliana* [29,30]. As studied, Pep1 is a 23-amino acid peptide that enhances resistance to a root pathogen, *Pythium irregulare*. Pep1 and its homologs (Pep2 to Pep7) are endogenous amplifiers of innate immunity of *Arabidopsis thaliana* that induce the transcription of defense-related genes and bind to PEPR1, a plasma membrane leucine-rich repeat (LRR) receptor kinase [31]. Our present results revealed that *T. atroviride* D16 PSF could gradually enhance the LRR protein kinase family protein and LRR receptor-like protein kinase PEPR2 in hairy roots of *S. miltiorrhiza*. The LRR receptor-like protein kinase PEPR2 might strengthen the ability of resisting *T. atroviride* D16 PSF, whereas the expression of probable LRR receptor-like serine/threonine-protein kinase At4g20940 decreased. CML42 are calcium-binding proteins that are thought to function as plant signal transduction elements, and were up-regulated and induced by *Spodoptera littoralis* in *Arabidopsis thaliana* followed by Ca^2+^ and phytohormone elevation [32,33]. In addition, CML42 enhanced the content of aliphatic glucosinolate and hyperactivated transcript accumulation of the jasmonic acid (JA)-responsive genes through the negative regulation of the jasmonate receptor Coronatine Insensitive1 (COI 1). As proteomics analysis revealed, this was expressed 1.7-fold higher in *S. miltiorrhiza* hairy root on the third day, which indicated *T. atroviride* D16 PSF may regulate Ca^2+^ and JA elevation. It has been reported that CML42 was not only involved in abiotic stress responses and insect herbivory defense, but also related to trichomes branching and endophytic fungi stimulation as a Ca^2+^ sensor, indicating CML42 is an important calcium-binding protein in plant growth and defense processes. Nucleoporin is a component of the nuclear pore complex, which has strongly attracted the attention of its involvement in hormonal and pathogen/symbiotic signaling [34,35]. Ca^2+^ signal transduction is important in the interaction between microorganisms and plants [36,37]. In the present study, as a key nucleoprotein, the Nup133/Nup155-like protein had a sharp rise on the third day as well as a substantial decrease in *S. miltiorrhiza* hairy root under the treatment of *T. atroviride* D16 PSF. The present results revealed that Nup133 are essential for mRNA export, and the Nup133/Nup155-like protein may regulate the symbiotic signaling transduction and *S. miltiorrhiza* response via mRNA export.

Cation-transporting ATPase is an enzyme protein widely found in biological membranes. Its structure and function is complex and plays an important regulatory role in the biological activities of cells [38]. PDE1 is an encoded P-type ATPase, which is required for the maintenance of phospholipid asymmetry in biological membranes, as membrane asymmetry may play a critical role in the development of infection hyphae by phytopathogenic fungi. The cation-transporting ATPase was up-regulated and induced by *T. atroviride* D16 PSF on the third day, and may provide a chance for interacting with *S. miltiorrhiza* hairy root. The 4-coumarate-Co-A ligase (4CL)-like proteins belong to the adenosine monophosphate (AMP)-binding domain-containing proteins family and widely exist in various plant species [39]. AMP-binding domain-containing 4CLs are critical enzymes in the phenylpropanoid metabolism pathway and drive the carbon flow from primary metabolism to different branches of secondary metabolism in plants [40]. Along with the tanshinones accumulation, the 4-coumarate-Co-A ligase (4CL)-like 10 protein were raised on the sixth day. In support, the 4-coumarate-Co-A ligase (4CL) like protein was up-regulated by *Magnaporthe oryzae* infection, which may be a defense-related AMP-binding protein (AMPBP) that is involved in the regulation of the defense response through salicylic acid (SA) and/or jasmonic acid (JA)/ ethylene (ET) signaling pathways. Phosphoprotein phosphatases (PPP) are present in all eukaryotic organisms, which is an ancient and important regulatory enzyme. The protein phosphatase 2Cs (PP2Cs) from various organisms have been implicated to act as negative modulators of protein kinase pathways involved in diverse environmental stress responses and developmental processes [41]. The Ser/Thr-specific phosphatases are metal-dependent enzymes divided into two major families: The PPP family, which includes protein phosphatases 1, 2A, and 2B (PP1, PP2A, PP2B/calcineurin); and the PPM family, which includes PP2C, which is a highly ABA-induced protein in guard cells once found in Arabidopsis and induced by endophyte fungi *T. atroviride* D16 PSF as well in this study. In addition, dual specificity protein phosphatases were significantly higher on the third day, referring to serine/threonine protein phosphatase and probable protein phosphatase related to protein phosphorylation [42]. *T. atroviride* D16 PSF induced high expression of protein phosphatase 2C and serine/threonine-protein phosphatase PP2A-2 through ABA signaling in *S. miltiorrhiza* hairy root. 12-oxophytodienoate reductase-like protein 1 is an enzyme in the jasmonic acid (JA) biosynthesis pathway [43], and the expression of 12-oxophytodienoate reductase-like protein 1 was gradually enhanced under the treatment of *T. atroviride* D16 PSF involved in the JA biosynthetic pathway. Superoxide dismutases (SODs) are involved in plant adaptive responses to biotic and abiotic stresses although the upstream signaling process which modulates their expression is not clearly understood [44]. Surprisingly, the level of SOD and peroxides were decreased at the treatment of *T. atroviride* D16 PSF, while plant overexpressing antioxidant enzymes had higher tolerance to external stress [45]. Phospholipid hydroperoxide glutathione peroxidase (PHGPX) is the principal enzymatic defense against oxidative destruction of biomembranes [46], and its major function appears to be the scavenging of phospholipid hydroperoxides, thereby protecting cell membranes from peroxidative damage. Gene expression analysis has shown an increase in the levels of PHGPX mRNA in several plant species undergoing different biotic and abiotic stresses, such as pathogen infections, high salt concentrations, exposure to heavy metals, mechanical stimulation, aluminum toxicity, seed germination, salt and osmotic stress, oxidative stress, and chilling stress [47]. PHGPXs may play dual roles as a redox transducer in addition to acting as a H_2_O_2_ scavenger under stress, thus PHGPX proteins may have different functions in plant cells, with some isoforms functioning in the signal transduction pathway, while others are involved in catalyzing the reduction of harmful products formed by ROS. The effect of various signaling molecules (salicylic acid, JA, ABA, ethylene) and certain protein phosphatase inhibitors (cantharidin and endothall) on the expression of the PHGPX gene in rice seedling leaves has demonstrated an up-regulation of the mRNA levels. These data suggest the role of this enzyme in the induction of defense mechanisms in plant cells subjected to oxidative damage, as a result of exposure to various environmental stresses, is by reducing the phospholipid hydroperoxides formed in the biomembranes.

## 5. Conclusions

Collectively, the analysis indicated that 89 differentially abundant proteins were involved mainly in protein synthesis, protein folding and degradation, biotic stress defense, photosynthesis, RNA process, signal transduction, and other functions. When induced by *T. atroviride* D16 PSF, *S. miltiorrhiza* hairy roots generally respond through elucidating the synthesis of tanshinones. These results provided valuable information for *T. atroviride* D16 PSF inducing tanshinones accumulation of *S. miltiorrhiza*. Characterization of PSF and proteomics provided an important bioinformatic resource for investigating mechanisms in inducing tanshinones accumulation.

## Figures and Tables

**Table 1:** The effects of D16 PSF and its monosaccharide constitutes on tanshinones accumulation on the fourteenth day.

Content (mg/g dw)	Control	60 mg/L PSF	60 mg/L Glucose	60 mg/L Mannose	60 mg/L Galactose
Dihydrotanshinone I	0.3548 ± 0.0084	1.0380 ± 0.0455 ***	0.4271 ± 0.0380	1.1641 ± 0.0133 ***	1.0559 ± 0.0367 ***
Cryptotanshinone	0.6421 ± 0.0211	1.9798 ± 0.0708 ***	1.2007 ± 0.0198 ***	0.6799 ± 0.0138	0.4209 ± 0.0099 ***
Tanshinone I	7.1378 ± 0.1455	11.6405 ± 0.1581 ***	9.3103 ± 0.1214 ***	10.7130 ± 0.1163 ***	7.4991 ± 0.1744
Tanshinone IIA	0.2321 ± 0.0022	0.4713 ± 0.0186 ***	0.3630 ± 0.0054 ***	0.2811 ± 0.0049 **	0.2639 ± 0.0056 *

D16 PSF means polysaccharide fraction from *T. atroviride* D16. Data were expressed as mean ± SD (*n* = 3), * *p* < 0.01, ** *p* < 0.01, *** *p* < 0.001, vs. control.

**Table 2:** The 10 KEGG pathways annotated by major differential proteins.

Pathway	Pathway Name	Protein Num.
ath00900	Terpenoid backbone biosynthesis	5
ath00010	Glycolysis/gluconeogenesis	3
ath00460	Cyanoamino acid metabolism	3
ath00940	Phenylpropanoid biosynthesis	3
ath03040	Spliceosome	3
ath00061	Fatty acid biosynthesis	2
ath00480	Glutathione metabolism	2
ath00590	Arachidonic acid metabolism	2
ath00630	Glyoxylate and dicarboxylate metabolism	2
ath03013	RNA transport	2

KEGG means Kyoto Encyclopedia of Genes and Genomes.

**Table 3:** The differential proteins of S. miltiorrhiza hairy roots after PSF treatment on third day and sixth day.

Accession	Signal Transduction Description	B/A	D/C	Accession	Signal Transduction Description	B/A	D/C
Leucine repeated cell proteins and receptors				O49289	Putative DEAD-box ATP-dependent RNA helicase 29	–	0.61
O22178	Leucine-rich repeat protein kinase family protein	1.54	2.09	Q9SCL3	PRE-MRNA SPLICING FACTOR SF2-like protein	–	1.63
Q9FZ59	Leucine-rich repeat receptor-like protein kinase PEPR2	1.70	3.38	Q56YD2	Nuclear protein-like	1.77	1.83
C0LGQ9	Probable LRR receptor-like serine/threonine-protein kinase At4g20940	–	0.65	Q9LZ82	AT5g04430/T32M21_30	–	3.06
Calcium ion and its related proteins				Q9SJK3	Protein argonaute 5	1.69	–
Q9SVG9	Calcium-binding protein CML42	1.72	–	Protein level			
Q9LT02	Probable cation-transporting ATPase	1.53	–	Q9C514	40S ribosomal protein S7-1	1.71	0.30
Q9SMT7	4-coumarate-CoA ligase-like 10	–	1.50	Q9SRT5	Putative translation initiation factor EIF-2B beta subunit, 3’ partial (Fragment)	0.57	0.64
Q8L7V5	AT3g52870/F8J2_40	–	0.51	F4K8L9	Chaperone DnaJ-domain containing protein	0.51	–
F4IGA4	Nucleoporin, Nup133/Nup155-like protein	0.41	12.75	Q9FGZ9	Ubiquitin-like protein 5	–	0.64
Protein phosphorylase				Q4FE45	E3 ubiquitin-protein ligase XBAT33	–	0.49
F4K7Q7	Dual specificity protein phosphatase family protein	2.04	–	Terpene biosynthesis			
Q07098	Serine/threonine-protein phosphatase PP2A-2 catalytic subunit	1.50	–	Q9SGY2	ATP-citrate synthase alpha chain protein 1	1.65	2.07
Q8RXV3	Probable protein phosphatase 2C 59	1.75	–	Q9FFT4	Pyruvate decarboxylase 2	–	1.72
Jasmonic acid synthesis				Q9XFS9	1-deoxy-D-xylulose 5-phosphate reductoisomerase, chloroplastic	1.54	–
Q8GYA3	Putative 12-oxophytodienoate reductase-like protein 1	1.64	2.96	Q94B35	4-hydroxy-3-methylbut-2-enyl diphosphate reductase, chloroplastic	1.54	–
Q593I2	At1g04380 (Fragment)	–	2.08	P54873	Hydroxymethylglutaryl-CoA synthase	–	1.60
Peroxidase				F4JNF1	Farnesyl diphosphate synthase 2	–	1.50
F4J504	Superoxide dismutase	0.64	–	F4HRA1	Cytochrome P450, family 76, subfamily C, polypeptide 5	–	2.24
O80912	Peroxidase 23	–	0.35	Carbohydrate synthesis and metabolism			
F4JS33	Peroxidase 50	–	0.49	Q9SGA8	UDP-glycosyltransferase 83A1	3.47	2.41
O48646	Probable phospholipid hydroperoxide glutathione peroxidase 6	1.52	–	Q9LXL5	Sucrose synthase 4	–	1.59
Signal transduction defense				Q9LJN4	Probable beta-D-xylosidase 5	–	0.56
P92948	Cell division cycle 5-like protein	–	0.60	Q9M076	6-phosphofructokinase 6	1.85	–
Oxidation and reduction				Q9LFR0	Alpha-mannosidase II	1.71	–
Oxidoreductase				Q85B88	Ribulose 1,5-bisphosphate carboxylase/oxygenase large chain (Fragment)	2.71	–
O80944	Aldo-keto reductase family 4 member C8	2.65	1.56	Q42306	Ribulose bisphosphate carboxylase small chain (Fragment)	–	12.36
O65621	Probable cinnamyl alcohol dehydrogenase 6	1.85	2.69	Transport protein			
Q84VY3	Acyl-[acyl-carrier-protein] desaturase 6, chloroplastic	–	0.66	Q56WK6	Patellin-1	0.65	0.57
Q9SA89	FAD-binding and BBE domain-containing protein	2.25	1.67	Q9SRU2	Auxin transport protein BIG	–	1.71
Q9SVG3	AT4G20840 protein	2.68	2.61	O65421	Vacuolar protein sorting-associated protein 28 homolog 1	2.40	–
Cytochrome				O24520	Non-symbiotic hemoglobin 1	1.92	5.16
Q9LIP3	Cytochrome P450 71B37	1.73	1.65	Q9M1H3	ABC transporter F family member 4	–	0.62
Q9CA60	Cytochrome P450 98A9	–	0.58	F4JL11	Importin subunit alpha	–	1.87
Q0WTJ5	Cytochrome P450 like protein (Fragment)	1.81	–	O23657	Ras-related protein RABC1	–	0.59
Q9LUD0	Cytochrome P450	–	1.51	C0Z3B2	AT1G22530 protein	–	0.60
Q9STH8	Cytochrome P450, family 706, subfamily A, polypeptide 7	1.52	2.09	Other functional protein			
F4HRA1	Cytochrome P450, family 76, subfamily C, polypeptide 5	–	2.24	P54967	Biotin synthase	–	0.38
O22912	Probable cytochrome c oxidase subunit 5C-1	1.72	–	O80575	6,7-dimethyl-8-ribityllumazine synthase, chloroplastic	–	0.64
Synthesis and metabolism of amino acids				P46011	Bifunctional nitrilase/nitrile hydratase NIT4	–	1.57
Q56WN1	Glutamine synthetase cytosolic isozyme 1-1	0.52	1.67	Q9C8S5	Chlorophyll A-B-binding protein 2, 5’ partial; 1-750 (Fragment)	1.64	–
Q9M9F1	Glutathione S-transferase U23	–	1.63	Q8LF21	Dynamin-related protein 1C	–	1.5
Q9FH05	Serine carboxypeptidase-like 42	0.66	–	Q0WRQ2	Enoyl-CoA hydratase like protein	–	1.52
Q94BN4	Putative methionine synthase	1.63	–	Q38939	GASA5	–	1.78
Q93Z70	Probable N-acetyl-gamma-glutamyl-phosphate reductase, chloroplastic	0.6	–	Q949M6	Putative uncharacterized protein At1g55040	0.48	–
Q9LFB5	AT5g01210/F7J8_190	1.73	–	Q0WPK8	Putative uncharacterized protein At1g72470	1.77	–
Protein synthesis and degradation				Unknown protein			
DNA level				Q94BQ9	Integral membrane HRF1-like protein	–	1.64
Q9FL33	DNA replication licensing factor MCM3 homolog	1.90	0.53	P83755	Photosystem Q(B) protein	–	1.97
Q05212	DNA-damage-repair/toleration protein DRT102	–	0.64	Q8GWN7	Putative uncharacterized protein At5g66090/K2A18_17	0.66	0.53
RNAlevel				Q8LB85	Putative uncharacterized protein	0.58	–
Q8H1D4	Double-stranded RNA-binding protein 4	–	2.28	Q9SBE3	T14P8.11 (Fragment)	3.91	5.23
Q9ZPY8	Transcription factor ABA-INDUCIBLE bHLH-TYPE	1.75	–	Q2HIQ2	At1g01170	–	0.65
Q9SKZ1	Transcription factor Pur-alpha 1	1.91	–				

Note: B/A and D/C are 3 and 6 days, respectively, dealing with the ratio of the amount of protein group and the blank group, 1.5 or higher to increase protein, 0.66 or less for cut protein, “–” indicates no difference. GASA5, GA-Stimulated in Arabidopsis; LRR, Leucine-rich repeat.

## References

[1] Wang L., Ma R., Liu C., Liu H., Zhu R., Guo S., Tang M., Li Y., Niu J., Fu M. (2017). *Salvia miltiorrhiza*: A potential red light to the development of cardiovascular diseases. Curr. Pharm. Des..

[2] Ren J., Fu L., Nile S.H., Zhang J., Kai G. (2019). *Salvia miltiorrhiza* in treating cardiovascular diseases: A review on its pharmacological and clinical applications. Front. Pharmacol..

[3] Jiang Z., Gao W., Huang L. (2019). Tanshinones, critical pharmacological components in *Salvia miltiorrhiza*. Front. Pharmacol..

[4] Zhang B., Zheng L.P., Wang J.W. (2012). Nitric oxide elicitation for secondary metabolite production in cultured plant cells. Appl. Microbiol. Biotechnol..

[5] Wang H., Yang X., Guo L., Zeng H., Qiu D. (2015). PeBL1, a novel protein elicitor from *Brevibacillus laterosporus* strain A60, activates defense responses and systemic resistance in *Nicotiana benthamiana*. Appl. Environ. Microbiol..

[6] Ming Q., Su C., Zheng C., Jia M., Zhang Q., Zhang H., Rahman K., Han T., Qin L. (2013). Elicitors from the endophytic fungus *Trichoderma atroviride* promote *Salvia miltiorrhiza* hairy root growth and tanshinone biosynthesis. J. Exp. Bot..

[7] Bohlmann H., Vignutelli A., Hilpert B., Miersch O., Wasternack C., Apel K. (1998). Wounding and chemicals induce expression of the *Arabidopsis thaliana* gene Thi2.1, encoding a fungal defense thionin, via the octadecanoid pathway. FEBS Lett..

[8] Smeekens S. (2000). Sugar- induced signal transduction in plants. Ann. Rev. Plant Physiol. Plant Mol. Biol..

[9] Wang H., Zhang X., Dong P., Luo Y., Cheng F. (2013). Extraction of polysaccharides from *Saccharomyces cerevisiae* and its immune enhancement activity. Int. J. Pharmacol..

[10] Chen F., Ren C.G., Zhou T., Wei Y.J., Dai C.C. (2016). A novel exopolysaccharide elicitor from endophytic fungus *Gilmaniella* sp. AL12 on volatile oils accumulation in *Atractylodes lancea*. Sci. Rep..

[11] Escribano J., Rubio A., Alvarez-Ortí M., Molina A., Fernández J.A. (2000). Purification and characterization of a mannan-binding lectin specifically expressed in corms of saffron plant (*Crocus sativus* L.). J. Agric. Food Chem..

[12] Schulz B., Rommert A.K., Dammann U., Aust H.J., Strack D. (1999). The endophyte-host interaction: A balanced antagonism?. Mycol. Res..

[13] Zhang H.Y., Lei G., Zhou H.W., He C., Liao J.L., Huang Y.J. (2017). Quantitative iTRAQ-based proteomic analysis of rice grains to assess high night temperature stress. Proteomics.

[14] Zhang Z., Zhou H., Yu Q., Li Y., Mendoza-Cózatl D.G., Qiu B., Liu P., Chen Q. (2017). Quantitative proteomics investigation of leaves from two *Sedum alfredii* (Crassulaceae) populations that differ in cadmium accumulation. Proteomics.

[15] Kaul S., Sharma T., Dhar M.K. (2016). “Omics” tools for better understanding the plant endophyte interactions. Front. Plant Sci..

[16] Alidrus A., Carpentier S.C., Ahmad M.T., Panis B., Mohamed Z. (2017). Elucidation of the compatible interaction between banana and *Meloidogyne incognitavia* high-throughput proteome profiling. PLoS ONE.

[17] Gadjeva M., Thiel S., Jensenius J.C. (2001). The mannan-binding-lectin pathway of the innate immune response. Curr. Opin. Immunol..

[18] Liu H., Guo Z., Gu F., Ke S., Sun D., Dong S., Liu W., Huang M., Xiao W., Yang G. (2016). 4-Coumarate-CoA ligase-Like gene OsAAE3 negatively mediates the rice blast resistance, floret development and lignin biosynthesis. Front. Plant Sci..

[19] Wasternack C. (2007). Jasmonates: An update on biosynthesis, signal transduction and action in plant stress response, growth and development. Ann. Bot..

[20] Song C., Zeng F., Wu F., Ma W., Zhang G. (2011). Proteomic analysis of nitrogen stress-responsive proteins in two rice cultivars differing in N utilization efficiency. J. Integr. Omics.

[21] He C.Y., Zhang G.Y., Zhang J.G., Duan A.G., Luo H.M. (2016). Physiological, biochemical, and proteome profiling reveals key pathways underlying the drought stress responses of *Hippophae rhamnoides*. Proteomics.

[22] Ruan S.L., Ma H.S., Wan S.H., Xin Y., Qian L.H., Tong J.X., Wang J. (2006). Advances in plant proteomics--I. Key techniques of proteome. Yi Chuan.

[23] Jiang Q., Li X., Niu F., Sun X., Hu Z., Zhang H. (2017). iTRAQ-based quantitative proteomic analysis of wheat roots in response to salt stress. Proteomics.

[24] Yang C., Xu L., Zhang N., Islam F., Song W., Hu L., Liu D., Xie X., Zhou W. (2017). iTRAQ-based proteomics of sunflower cultivars differing in resistance to parasitic weed *Orobanche cumana*. Proteomics.

[25] Kambiranda D., Katam R., Basha S.M., Siebert S. (2014). iTRAQ-based quantitative proteomics of developing and ripening muscadine grape berry. J. Proteome Res..

[26] Zheng B.B., Fang Y.N., Pan Z.Y., Sun L., Deng X.X., Grosser J.W., Guo W.W. (2014). iTRAQ-based quantitative proteomics analysis revealed alterations of carbohydrate metabolism pathways and mitochondrial proteins in a male sterile cybrid pummelo. J. Proteome Res..

[27] Keerthisinghe S., Nadeau J.A., Lucas J.R., Nakagawa T., Sack F.D. (2015). The Arabidopsis leucine-rich repeat receptor-like kinase MUSTACHES enforces stomatal bilateral symmetry in Arabidopsis. Plant J. Cell Mol. Biol..

[28] Zhou F., Yong G., Qiu L.J. (2016). Genome-wide identification and evolutionary analysis of leucine-rich repeat receptor-like protein kinase genes in soybean. BMC Plant Biol..

[29] Shahollari B., Vadassery J., Varma A., Oelmüller R. (2007). A leucine-rich repeat protein is required for growth promotion and enhanced seed production mediated by the endophytic fungus *Piriformospora indica* in *Arabidopsis thaliana*. Plant J..

[30] Park S.J., Moon J.C., Yong C.P., Kim J.H., Dong S.K., Jang C.S. (2014). Molecular dissection of the response of a rice leucine-rich repeat receptor-like kinase (LRR-RLK) gene to abiotic stresses. J. Plant Physiol..

[31] Yamaguchi Y., Huffaker A., Bryan A.C., Tax F.E., Ryan C.A. (2010). PEPR2 is a second receptor for the Pep1 and Pep2 peptides and contributes to defense responses in Arabidopsis. Plant Cell.

[32] Stephanie D., David C., Polly L., Steven P.S., Snedden W.A. (2009). The calmodulin-related calcium sensor CML42 plays a role in trichome branching. J. Biol. Chem..

[33] Vadassery J., Reichelt M., Hause B., Gershenzon J., Boland W., Mithöfer A. (2012). CML42-mediated calcium signaling coordinates responses to spodoptera herbivory and abiotic stresses in Arabidopsis. Plant physiol..

[34] Vasu S., Shah S., Orjalo A., Park M., Fischer W., Forbes D. (2001). Novel vertebrate nucleoporins Nup133 and Nup160 play a role in mRNA export. J. Cell Biol..

[35] Martin B., Thoe F.A., Doan-Trung L., Hervé S., Isabelle G., Isabelle C. (2016). Reduced expression of AtNUP62 nucleoporin gene affects auxin response inArabidopsis. BMC Plant Biol..

[36] Wenping H., Yuan Z., Jie S., Lijun Z., Zhezhi W. (2011). De novo transcriptome sequencing in *Salvia miltiorrhiza* to identify genes involved in the biosynthesis of active ingredients. Genomics.

[37] Snedden W.A., Fromm H. (2001). Calmodulin as a versatile calcium signal transducer in plants. New Phytol..

[38] Balhadère P.V., Talbot N.J. (2001). PDE1 encodes a P-type ATPase involved in appressorium-mediated plant infection by the rice blast fungus *Magnaporthe grisea*. Plant Cell.

[39] Zhang X.C., Yu X., Zhang H.J., Song F.M. (2009). Molecular characterization of a defense-related AMP-binding protein gene, OsBIABP1, from rice. Biomed Biotechnol..

[40] Soltani B.M., Ehlting J., Douglas C.J. (2006). Genetic analysis and epigenetic silencing of At4CL1 and At4CL2 expression in transgenic Arabidopsis. Biotechnol. J..

[41] Xue T., Wang D., Zhang S., Ehlting J., Ni F., Jakab S., Zheng C., Zhong Y. (2008). Genome-wide and expression analysis of protein phosphatase 2C in rice and Arabidopsis. BMC Genom..

[42] Cohen P. (1989). The structure and regulation of protein phosphatases. Ann. Rev. Biochem..

[43] Sanders P.M., Lee P.Y., Biesgen C., Boone J.D., Beals T.P., Weiler E.W., Goldberg R.B. (2000). The arabidopsis DELAYED DEHISCENCE1 gene encodes an enzyme in the jasmonic acid synthesis pathway. Plant Cell.

[44] Xing Y., Chen W., Jia W., Zhang J. (2015). Mitogen-activated protein kinase kinase 5 (MKK5)-mediated signalling cascade regulates expression of iron superoxide dismutase gene in Arabidopsis under salinity stress. J. Exp. Bot..

[45] Novo-Uzal E., Gutiérrez J., Martínez-Cortés T., Pomar F. (2014). Molecular cloning of two novel peroxidases and their response to salt stress and salicylic acid in the living fossil Ginkgo biloba. Ann. Bot..

[46] Chen S., Vaghchhipawala Z., Li W., Asard H., Dickman M.B. (2004). Tomato phospholipid hydroperoxide glutathione peroxidase inhibits cell death induced by Bax and oxidative stresses in yeast and plants. Plant Physiol..

[47] Jain P., Bhatla S.C. (2014). Signaling role of phospholipid hydroperoxide glutathione peroxidase (PHGPX) accompanying sensing of NaCl stress in etiolated sunflower seedling cotyledons. Plant Signal. Behav..

